# THE S.O.C. MODEL OF AGING IN DOCUMENTARY FILMS: POSITIVE ADAPTATION TO AGE-RELATED LOSS IN THEORY AND EVERYDAY LIFE

**DOI:** 10.1093/geroni/igz038.1518

**Published:** 2019-11-08

**Authors:** Rick Scheidt

**Affiliations:** Kansas State University, Manhattan, Kansas, United States

## Abstract

There is overwhelming evidence that “pop culture” depictions of age-related losses are primarily negative, ignoring positive adaptive experiences associated with the second half of life. Unfortunately, film as an entertainment medium often creates and reinforces this negative status quo. This presentation describes the usefulness of the Baltes and Baltes S.O.C. Model for offsetting losses – via narrowing and revision of goal priorities (Selection), locating and enhancing resources to achieve positive outcomes (Optimization), and using these to increase one’s personal limits and reserve capacities (Compensation). In addition, positive “S.O.C. Solutions” (Spiehs, 2018) are illustrated for everyday loss scenarios within four new documentary films. These include positive adaptations to four loss domains – personal autonomy (driving), physical capacity (sexual responsiveness), psychological well-being (loneliness and belonging), and environmental destruction (place dependency). Annotated sources will be made available.

